# A case of hepatic venous outflow obstruction caused by migration of the remnant liver into the subphrenic space after extended posterior sectionectomy of the liver

**DOI:** 10.1016/j.ijscr.2020.09.203

**Published:** 2020-10-06

**Authors:** Hiroki Kanno, Atsushi Yoshida, Yuichi Goto, Toru Hisaka, Yoshito Akagi, Koji Okuda

**Affiliations:** Department of Surgery, Kurume University School of Medicine, Kurume, Japan

**Keywords:** ALT, alanine aminotransferase, AST, aspartate aminotransferase, CT, computed tomography, HVOO, hepatic venous outflow obstruction, MHV, middle hepatic vein, POD, post operative-day, US, ultrasonography, T.bil, total bilirubin, Hepatic venous outflow obstruction, Hepatectomy, Repositioning

## Abstract

**Introduction:**

Hepatic venous outflow obstruction (HVOO) is a rare complication of hepatectomy. We report a case of HVOO caused by remnant liver migration into the subphrenic space after hepatectomy, which was successfully managed by repositioning of the remnant liver.

**Presentation of case:**

A 55-year-old Japanese man was diagnosed with a liver tumor on ultrasound. Contrast-enhanced CT revealed early enhancement in the arterial phase, followed by a washout in the late phase. Preoperative diagnosis was hepatocellular carcinoma, and hand-assisted laparoscopic extended posterior sectionectomy was performed. On postoperative day 1, middle hepatic vein (MHV) flow was not detected on ultrasound, and the portal flow was hepatofugal. CT during arterial portography revealed absence of the portal flow to the medial and anterior sections, and remnant liver migration into the subphrenic space. Therefore, we suspected that HVOO was caused by the remnant liver migration and performed redo laparotomy to reposition the remnant liver with suturing of the falciform ligament to the anterior abdominal wall. Postoperatively, contrast-enhanced CT demonstrated that the remnant liver remained in the anatomical position, and the medial and anterior sections were well enhanced.

**Discussion:**

HVOO might occur irrespective of whether the left triangular ligament is preserved. We believe that it is necessary to fix the remnant liver to the abdominal wall in cases with poor venous blood flow confirmed by intraoperative ultrasound. If kinking of the hepatic vein persists, stent insertion should be performed.

**Conclusion:**

HVOO after hepatectomy is rare but potentially fatal, and prevention and countermeasures should be discussed.

## Highlights

• Hepatic venous outflow obstruction (HVOO) is a rare complication of hepatectomy.
• HVOO might occur irrespective of whether the left triangular ligament is divided.
• The treatment for HVOO is repositioning and/or stenting into the hepatic vein.

## 1 Introduction

Hepatic venous outflow obstruction (HVOO) is caused by stenosis or occlusion of either the hepatic vein or the inferior vena cava, leading to hepatic congestion and liver failure. HVOO is a lethal complication in living donor liver transplantation (LDLT); its incidence is 3.3%–12.5% in LDLT [1,2], but it is rare (0.1%) in hepatectomy [3]. Herein, we report a case of HVOO caused by migration of the remnant liver into the right subphrenic space after extended posterior sectionectomy of the liver, which was successfully managed by repositioning of the remnant liver with suturing of the falciform ligament to the anterior abdominal wall. This work has been reported in line with the SCARE criteria [4].

## 2 Presentation of case

**Fig. 1:**
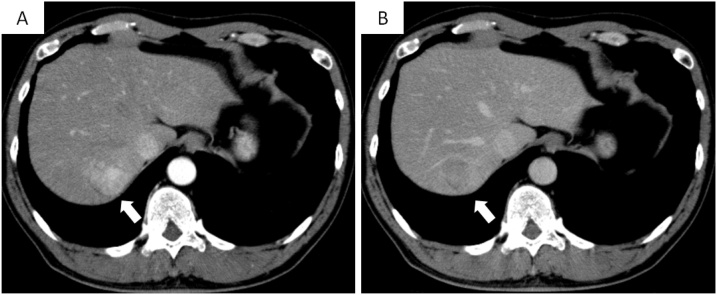
Tumor is enhanced in the arterial phase (A), followed by a washout in the late phase (B) on contrast-enhanced computed tomography (arrow).

**Fig. 2:**
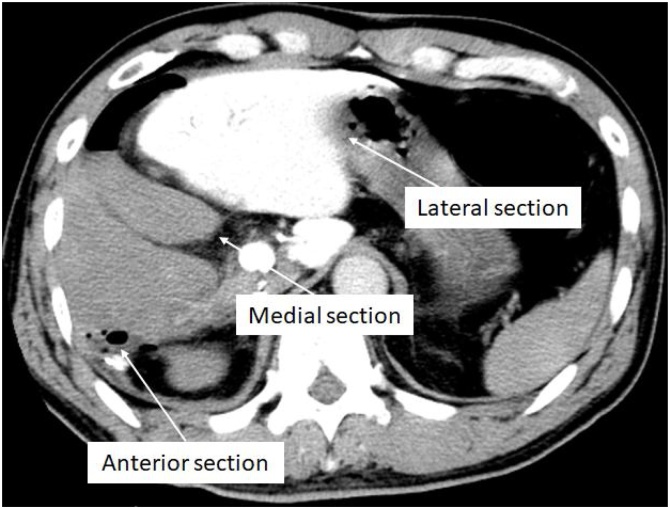
Only the lateral section is enhanced. The medial and anterior sections are not enhanced on computed tomography during arterial portography. The remnant liver migrates into the right subphrenic space.

**Fig. 3:**
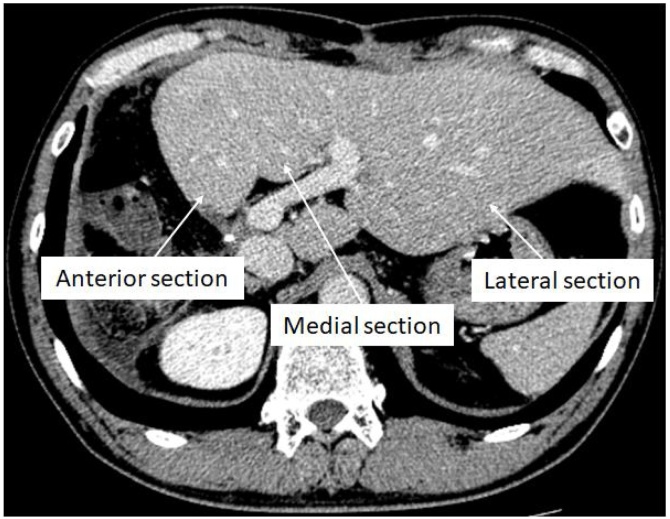
The remnant liver remains in the anatomical position, and medial and anterior sections are well enhanced on computed tomography.

A 55-year-old Japanese man was diagnosed with a liver tumor on abdominal ultrasound (US) performed during his annual medical check-up. He had a history of appendicitis and fatty liver. On physical examination, there were no abnormalities. Laboratory tests showed no remarkable abnormalities: aspartate aminotransferase (AST) 23 U/L, alanine aminotransferase (ALT) 37 U/L, alkaline phosphatase 165 U/L, γ-glutamyltransferase 36 U/L, total bilirubin (T.bil) 0.9 mg/dL, albumin 4.70 g/dL, platelet count 19.8 × 10^4^ /μL, and prothrombin time 105%. The Child–Pugh score was 5. Hepatitis virus serological markers were absent. The tumor marker protein-induced vitamin K absence-II was elevated (315 mAU/mL). Other tumor markers were within normal range: α-fetoprotein 5.0 ng/mL, carcinoembryonic antigen 1.3 ng/mL, and carbohydrate antigen 19-9 25.0 U/mL. Abdominal US revealed a round hypoechoic nodule in segment 7, measuring 44 mm in diameter. Contrast-enhanced computed tomography (CT) revealed early enhancement in the arterial phase, followed by a washout in the late phase (Fig. 1). On magnetic resonance imaging, the nodule demonstrated high signal intensity on T2-weighted images, low signal intensity on T1-weighted images, and high signal intensity on diffusion-weighted images. The enhancement pattern was almost the same as the CT findings. Preoperative diagnosis was hepatocellular carcinoma, and hand-assisted laparoscopic extended posterior sectionectomy with preservation of the left triangular ligament was performed. Operation time was 660 min, and blood loss was 495 mL. On postoperative day (POD) 1, liver enzymes were elevated: AST 681 U/L, ALT 931 U/L, and T.bil 3.7 mg/dL. Although abdominal US confirmed proper hepatic arterial flow and there was no portal vein thrombosis, blood flow in the middle hepatic vein (MHV) was not detected, and the portal flow was hepatofugal. On POD2, ALT and T.bil levels increased (ALT 952 U/L, T.bil 5.21 mg/dL), and the postoperative course was different from that of usual liver resection. Subsequently, CT during arterial portography was performed, which revealed the absence of portal flow to the medial and anterior sections (Fig. 2). Additionally, the remnant liver had dislocated to the right subphrenic space. Therefore, we suspected HVOO caused by migration of the remnant liver and performed a redo laparotomy for repositioning of the remnant liver to the anatomical position on POD3. Operative findings revealed that the remnant liver had migrated into the right subphrenic space, and the anterior section was congestive. After pulling up the falciform ligament, the congestion of the anterior section disappeared. We sutured the falciform ligament to the anterior abdominal wall. MHV flow and portal flow to the anterior section were clearly confirmed on US after the procedures. The postoperative course was uneventful. The serum levels of liver enzymes promptly decreased, and each blood flow was well confirmed on US. He was discharged on POD 19. Contrast-enhanced CT on POD 54 demonstrated that the remnant liver remained in the anatomical position in the abdominal cavity, and the medial and anterior sections were well enhanced (Fig. 3). He has survived for more than 10 years without recurrence.

## 3 Discussion

The incidence of HVOO after hepatectomy (0.1%) is lower than that after LDLT (3.3%–12.5%). Some researchers report that this is because the preservation of the supporting tissues such as the left triangular ligament and the main trunk of MHV prevents migration of the remnant liver into the subphrenic space [5,6]. However, Ogata et al. reported that preserving the left triangular ligament was not sufficient to maintain the hepatic venous outflow after right hepatectomy. In their case study, there was no significant difference in the hepatic venous outflow between the cases with preserved left triangular ligament and those without it [7]. Additionally, although the left triangular ligament and the main trunk of MHV were preserved in the present case and some cases [3,8,9], migration of the remnant liver was noted, suggesting that HVOO occurs even when the supporting tissues exist.

We searched the PubMed database using the keywords “hepatectomy” and “outflow obstruction.” This search yielded 15 cases of HVOO after hepatectomy; these cases are summarized in Table 1. Van et al. reported that a tumor size of >180 mm was considered a risk factor for HVOO [3]; however, HVOO also occurred in cases with relatively smaller tumors such as that in the present case and case 8, measuring 44 mm and 30 mm in diameter, respectively [10]. Factors apart from tumor diameter might be associated with HVOO. Surgeries performed were as follows: 8 right hepatectomies, 4 extended right hepatectomies, 2 right trisectionectomies, and 1 extended posterior sectionectomy. All patients underwent right hepatectomy; in such patients, the wide vacant space on the right subphrenic area after hepatectomy might induce migration of the liver. The time from the initial operation to the intervention ranged from 0 to 240 days. Chronic cases (case 5 and case 8), i.e., those with onset after > 150 days, were owing to inadequate hepatic parenchymal regeneration. Hypertrophic hepatic parenchyma caused migration of the remnant liver and kinking of the hepatic vein. Treatments for HVOO were repositioning, interventional radiology, and both in 6, 6, and 1 case, respectively. Despite the fact that fixation to the anterior abdominal wall was performed in the initial operation, HVOO occurred in 2 cases [11,12]. In those cases, stent insertion was required. In one case, only ballooning was performed; however, the effect of ballooning was temporary, and additional stenting was required [10]. Thus, stenting is preferable to ballooning. In one case of trauma, the patient was managed by open abdomen owing to bowel edema, which hindered the fixation of the remnant liver to the anterior abdominal wall. In this case, a saline bag was used to prevent the remnant liver from migrating into the subphrenic space [13].

**Table 1:** Characteristics of 15 cases with hepatic venous outflow obstruction.

Case	Age	Sex	Diagnosis	History of abdominal surgery	Tumor size (mm)	Initial operation	Triangular ligament	Time from operation to intervention (Day)	Treatment for HVOO	Author
1	29	F	Metastatic	ND	80	Right hepatectomy	Preserved	2	Stenting	Van Ha
2	64	M	HCC	None	90	Extended right hepatectomy	ND	18	Stenting	Lhuaire M
3	23	M	Trauma	ND	–	Right hepatectomy	ND	6	Saline bag	Ariche A
4	67	M	HCC	None	180	Right hepatectomy	Preserved	1	Repositioning	Sato N
5	41	M	HCC	ND	Huge†	Right trisectionectomy	Divided	150	Balloon	Imai D
6	76	F	HCC	ND	180	Extended right hepatectomy	Divided	1	Repositioning, Stenting	Di Domenico
7	46	F	Metastatic	ND	ND	Right hepatectomy	Preserved	15	Stenting	Wang JK
8	46	M	CCC	ND	30	Extended right hepatectomy	ND	240	Stenting	Ninomiya M
9	15	M	HCC	ND	210	Extended right hepatectomy	ND	13	Stenting	Benesch M
10	37	M	Inflammatory pseudotumor	ND	Huge†	Right trisectionectomy	ND	0	Repositioning	Poon RT
11	56	M	Metastatic	APR	ND	Right hepatectomy	Divided	32	Drainage	Nakashima K
12	75	M	HCC	ND	ND	Right hepatectomy	Divided	7	Repositioning	Pitre J
13	55	M	Metastatic	ND	Huge†	Right hepatectomy	Divided	1	Repositioning	Pitre J
14	8	F	Wilms tumor of the kidney	ND	–	Right hepatectomy	ND	70	Repositioning	Sequeira FW
15	55	M	HCC	Appendectomy	44	Extended posterior sectionectomy	Preserved	2	Repositioning	Present case

†: Tumor diameter was not mentioned.

APR: abdominoperineal resection, HVOO: hepatic venous outflow obstruction, ND: not described.

HVOO might occur irrespective of whether the left triangular ligament is preserved. Some researchers recommend routine fixation of the remnant liver to the anatomical position using the falciform ligament for the prevention and treatment of HVOO. In the present case, repositioning was effective. We believe that it is necessary to fix the remnant liver to the abdominal wall in cases with poor venous blood flow confirmed by intraoperative US. If kinking of the hepatic vein persists, stent insertion should be performed.

## 4 Conclusion

After right hepatectomy, the remnant liver tends to spontaneously rotate around the inferior vena cava, which can cause HVOO owing to kinking of the hepatic vein. In the present case, HVOO was successfully managed by repositioning. However, in some cases in which repositioning fails to prevent or treat HVOO, stenting is required.

## Declaration of Competing Interest

The authors declare that they have no competing interest.

## Funding

The authors declare that this work was not supported by any grants or funding.

## Ethical approval

Ethical approval was obtained from the Ethics Committee of Kurume University (No. 2020-038).

## Consent

Written informed consent was obtained from the patient for publication of this case report and accompanying images. A copy of the written consent is available for review by the Editor-in-Chief of this journal on request.

## Author contribution

HK drafted the manuscript. KO and YA supervised the study. AY, YG, and TH performed perioperative management of the patient.

## Registration of research studies

Not applicable.

## Guarantor

Hiroki Kanno.

## Provenance and peer review

Not commissioned, externally peer-reviewed.
